# Maternal COVID-19 Disease and COVID-19 Immunization

**DOI:** 10.7759/cureus.28328

**Published:** 2022-08-23

**Authors:** Amala Sunder, Bessy Varghese, Omer Taha, Mohamed S Keshta, Ameena Khalid Bughamar, Enas Nadir Abelhamid Mohamed, Yusra Mirghani Aljailani Fadhulalla, Basma Darwish

**Affiliations:** 1 Obstetrics and Gynecology, Bahrain Defense Force Hospital, West Riffa, BHR; 2 Medicine, Royal College of Surgeons in Ireland - Bahrain, Busaiteen, BHR; 3 Orthopedics, Royal College of Surgeons in Ireland - Bahrain, Busaiteen, BHR

**Keywords:** umbilical cord blood, vertical transmission, gestational age, maternal age, immune response, covid-19 vaccination, covid-19 infection, anti sars antibodies

## Abstract

Aim

This study aimed to evaluate the immune response and vertical transmission of anti-severe acute respiratory syndrome (SARS) antibodies in vaccinated, expectant mothers infected with coronavirus disease 2019 (COVID-19) and to study the sequelae.

Study design

This was a retrospective study of pregnant women conducted at Bahrain Defense Force Hospital from March 2021 to September 2021. The study population was divided into two groups: group 1 was vaccinated with Sinopharm or Pfizer/BioNTech during pregnancy and never infected with COVID-19. Group 2 was unvaccinated and had been infected with COVID-19. Immune responses such as anti-nucleocapsid (anti-N) and anti-spike (anti-S) from paired samples of maternal and umbilical cord blood were measured with Elecsys immunoassay (Roche Holding AG: Basel, Switzerland) at the time of delivery. Obstetric complications such as preterm labor, preeclampsia, and stillbirth were assessed. Analysis was performed using SPSS version 26.0 (IBM Corp: Armonk, NY) and Minitab version 18 (Minitab, LLC: State College, PA). A p-value of less than 0.05 was considered statistically significant.

Results

The study included 90 vaccinated and 90 COVID-19-recovered pregnant women. Matched samples were available for 80 vaccinated and 74 COVID-19-recovered women. Group 1 had significantly higher levels of anti-S for both the mother and the cord blood and a significantly higher transfer ratio of anti-S. Group 2 had higher levels of anti-N. In group 1, the paired sample titer of anti-S had a weak negative correlation with maternal age whereas, in group 2, the mother’s anti-N had a weak positive correlation with age. Antibodies of COVID-19-recovered mothers and cord blood had a moderate negative correlation with gestational age, except for the mother’s anti-N. In group 1, the transfer ratio of anti-N and anti-S had a statistically significant association with gestational age. Preterm delivery had a high prevalence of anti-transfer ratios of <1, and delivery at >37 weeks had a high prevalence of ≥1. In group 2, 90% of preterm deliveries had transfer ratios of anti-S <1. The latency period of the COVID-19 group had a statistically significant association with the antibody transfer ratio. An interval of less than 100 days had a high prevalence in the ratio of <1. An interval of more than 100 days had a high prevalence in the ratio of* ≥*1*. *There was no significant latency period in group 1. Group 1 had a 75% prevalence of an anti-S transfer ratio *≥*1 with a birth weight of >3500 g; group 2 had no significance in birth weight. We did not find significance in the sequelae of morbidities in either group.

Conclusion

The production of the antibody N in the COVID-19-infected and antibody S in the vaccinated pregnant women as well as the vertical transmission of antibodies was efficacious. Significant variation was found regarding maternal age in both groups. The transfer ratio of the antibodies in the vaccinated and COVID-19-recovered women was significantly higher in terms of babies of the vaccinated and the infected population. The transfer ratios were distinct according to the latency period and birth weight of the infants.

## Introduction

The severity of coronavirus disease 2019 (COVID-19) increases during pregnancy, and the virus has been associated with maternal and fetal morbidities such as critical care admission during COVID-19 infection, venous thromboembolism, preeclampsia, and preterm labor [1-3]. The COVID-19 vaccine is a promising protective approach to reducing the incidence of morbidities during pregnancy. Currently, the COVID-19 vaccines are recommended for pregnant women worldwide; the benefits are greater than the risks. The UK’s Joint Committee on Vaccination and Immunization recommends vaccination for all pregnant women before 27 weeks because disease severity increases after this period [4]. The approved vaccines are not live, so they cannot cause actual disease in the women or the fetuses [5]. Quantifying the immune response of the vaccinated vs. the infected pregnant women as well as vertical transmission is important in understanding how to protect against infection. Comparing the immune response of the COVID-19-infected and vaccinating pregnant women is crucial for future recommendations for the pregnant population.

## Materials and methods

The study was conducted in our tertiary center, Bahrain Defense Force Hospital, from March to September 2021. It began with receiving informed consent and included 180 pregnant women. The study design was approved by our research center’s ethical committee and the national COVID-19 clinical research team. The cohort was divided into group 1, which included 80 vaccinated, and non-infected pregnant women, and group 2, which included 74 non-vaccinated, and COVID-19-recovered pregnant women. Infection was excluded by nasopharyngeal samples using the polymerase chain reaction (PCR) or SARS-CoV-2 GeneXpert before checking antibody levels. Twenty-five people were not included in our analysis of immune responses because matched sera were not available. Demographic data were assessed in detail. Evaluation of computerized records was done confidentially. All data were rendered anonymous. The cohort was interviewed about the types of vaccines they had received, their gestational ages at their first and second doses, and their COVID-19 diagnoses. The gestational age at delivery and interval of immunoassay were calculated. Antibodies from both groups in response to the spike protein (S) and nucleocapsid protein (N) and their vertical transmission were assessed using the mother’s serum at the time of delivery. Interval frequencies of antibodies from the second vaccine dose and from COVID-19 infection were calculated. The association of immune response with maternal factors, including maternal age, BMI, gestational period, gestational age at delivery, latency period, and transfer ratio, was calculated and compared between the groups. The transfer ratio level was divided; and the association with maternal age, BMI, gestational age at delivery, gender of the baby, birth weight of the baby, and latency period was checked in both groups. Cohorts were continuously assessed for sequelae following vaccination and COVID-19 infection. Both populations were assessed throughout pregnancy to identify complications.

Inclusion criteria

Group 1 included vaccinated pregnant women who received two doses during pregnancy and had matched paired samples of umbilical cord blood. Group 2 included women who were infected with COVID-19 and had paired samples. In both groups, participants with and without matched samples were included in the analysis of sequelae.

Exclusion criteria

To ensure uniformity, individuals in group 1 who received single dose or booster doses of vaccination were excluded. To reduce bias, anyone who was infected with COVID-19 at any point during or before pregnancy or got vaccinated before pregnancy was also excluded. Group 2 excluded women vaccinated before and during pregnancy. The population without matched sera was excluded from antibody analysis.

Statistical analysis

Continuous variables were represented as mean±standard deviation, or median (first quartile, third quartile), whereas categorical variables were represented as frequencies and percentages. Depending on the data requirements, independent t-test and Mann-Whitney U test were used to compare the characteristics of the vaccinated group and the COVID-19 infection group. Pearson and Spearman correlations were used to assess the relationship between maternal factors, mother's antibodies, cord blood antibodies, and antibody transfer ratio. To assess associations between categorical variables, chi-square and Fisher's exact tests were used. SPSS version 26.0 (IBM Corp: Armonk, NY) and Minitab version 18 (Minitab, LLC: State College, PA) software were used to conduct all analyses. A p-value of less than 0.05 was considered statistically significant.

## Results

The analysis included a total of 154 women, 80 of whom were vaccinated while pregnant and the other 74 were infected with COVID-19 while pregnant. The patients' age ranged from 18 to 42 years, with four vaccinated mothers and 21 COVID-19 patients having comorbidities. Characteristics of the vaccinated and the infected groups are summarized in Table 1.

**Table 1:** Demographics characteristics and association of vaccinated and COVID-19-infected mothers. *Significant p-value <0.05 level. **Significant p-value <0.01 level. P-value was calculated using independent t-test or Mann-Whitney test as appropriate, antibodies interval for vaccinated group is from the second dose of vaccination. COVID-19: coronavirus disease 2019

Variables		Vaccinated (n=80)	COVID-19 infected (n=74)	p-Value
Mother’s age (mean±SD)		29.2±5.843	28.74±5.967	0.632
BMI (mean±SD)		31.89±5.967	30.189±5.446	0.073
Baby’s weight (g) (mean±SD)		3147.54±628.151	3215.54±495.47	0.459
Gestational age (weeks) median (Q1, Q3)		29 (24, 33)	26 (21, 31)	0.034*
Delivery gestational age (weeks) median (Q1, Q3)		39 (37, 40)	39 (37, 40)	0.827
Ab levels (mother) median (Q1, Q3)	Antibody N (COI)	0.61 (0.09, 3.25)	19.6 (5.64, 50.9)	<0.01**
	Antibody S (U/mL)	380 (65.35, 1442.5)	69 (23.4, 191)	<0.01**
Ab levels (baby) median (Q1, Q3)	Antibody N (COI)	0.81 (0.09, 3.35)	18.05 (3.73, 63.9)	<0.01**
	Antibody S (U/mL)	429.5 (43.2, 1610)	60.35 (15.7, 179)	<0.01**
Transfer ratio median (Q1, Q3)	Antibody N	1 (0.81, 1.29)	0.99 (0.76, 1.33)	0.960
	Antibody S	1.23 (0.68,1.6)	0.88 (0.68, 1.08)	0.013*
Interval (days) median (Q1, Q3)		79 (55, 117)	87 (53, 117)	0.939

The median gestational age for vaccination was 29 weeks. The gestational age for COVID-19 infection was 26 weeks on average. The antibodies N and S of mothers and babies differed statistically between vaccinated and COVID-19-infected patients, with the vaccinated group having significantly higher levels of antibody S for both mothers and babies and the COVID-19-infected group having higher levels of antibody N. Transfer ratio of antibody S had a significant difference.

Maternal factors correlation with mother’s and cord blood antibodies of vaccinated group and COVID-19-infected group are presented in Table 2. In vaccinated group, mother’s and cord blood antibody S had a weak negative correlation with age. Mother’s antibody N from COVID-19 infected group had a weak positive correlation with age, all antibodies of COVID-19-infected mothers and cord blood had a moderate negative correlation with gestational age except mother’s antibody N.

**Table 2:** Correlation between maternal factors and vaccinated mother's and cord's antibodies, and maternal factors and COVID-19-infected mother's and cord’s antibodies. *Significant p-value < 0.05 level. **Significant p-value < 0.01 level. P-value was calculated using Pearson correlation or Spearman correlation as appropriate. COVID-19: coronavirus disease 2019

Variables		Mother antibody N	Mother antibody S		Cord antibody N	Cord antibody S
Vaccinated mothers and cord blood	Mother’s age	r = -0.034		r = -0.295	r = 0.019	r = -0.275
		p = 0.769		p = 0.01*	p = 0.877	p = 0.022*
	BMI	r = 0.127		r = -0.165	r = 0.144	r = -0.093
		p = 0.288		p = 0.165	p = 0.251	p = 0.460
	Baby’s weight	r = 0.158		r = -0.047	r = 0.153	r = -0.027
		p = 0.174		p = 0.688	p = 0.206	p = 0.826
	Gestational age	rs = 0.041		rs = 0.120	rs = 0.045	rs = 0.181
		p = 0.726		p = 0.307	p = 0.713	p = 0.137
	Gestational age at delivery	rs = -0.028		rs = -0.091	rs = -0.054	rs = 0.015
		p = 0.812		p = 0.434	p = 0.656	p = 0.902
	Interval	rs = -0.100		rs = -0.130	rs = -0.120	rs = -0.150
		p = 0.392		p = 0.266	p =0.325	p = 0.217
COVID-19-infected mothers and cord blood	Mother’s age	r = 0.252		r = 0.193	r = 0.182	r = 0.210
		p = 0.03*		p = 0.100	p = 0.121	p = 0.072
	BMI	r = 0.146		r = 0.002	r = 0.154	r = 0.178
		p = 0.215		p = 0.989	p = 0.190	p = 0.128
	Baby’s weight	r = -0.110		r = 0.118	r = -0.096	r = 0.105
		p = 0.349		p = 0.316	p = 0.417	p = 0.371
	Gestational age	rs = -0.120		rs = -0.330	rs = -0.358	rs = -0.474
		p = 0.328		p = 0.006**	p = 0.003**	p ≤ 0.01**
	Gestational age at delivery	rs = -0.257		rs = -0.227	rs = -0.137	rs = -0.138
		p = 0.027*		p = 0.052	p = 0.244	p = 0.240
	Interval	rs = -0.024		rs = 0.244	rs = 0.244	rs = 0.421
		p = 0.839		p = 0.036*	p = 0.036*	p ≤ 0.01**

Correlation between maternal factors and antibodies transfer ratio of the vaccinated and COVID-19-infected groups is represented in Table 3. Transfer ratio of antibodies N and S from COVID-19-recovered patients had a strong negative correlation with gestational age and a positive correlation with the interval, whereas antibody N transfer ratio from the COVID-19 group and anti-S and N of vaccinated group had a significant correlation with gestational age at delivery (Table 3).

**Table 3:** Correlation between maternal factors with vaccinated and positive COVID-19 antibodies transfer ratio. *Significant p-value < 0.05 level. **Significant p-value < 0.01 level. Of note, r and rs are correlations that show the strength of the relations, where "r" for Pearson correlation and "rs" for Spearman correlation. And, the p-value was calculated using Pearson correlation or Spearman correlation as appropriate. COVID-19: coronavirus disease 2019; anti-N: anti-nucleocapsid; anti-S: anti-spike

Variables	Vaccinated		Positive COVID-19 infection	
	Anti-N transfer ratio	Anti-S transfer ratio	Anti-N transfer ratio	Anti-S transfer ratio
Age	r = -0.048	r = -0.001	r = -0.183	r = -0.190
	p = 0.678	p = 0.993	p = 0.118	p = 0.104
BMI	r = -0.143	r = -0.108	r = -0.009	r = 0.123
	p = 0.231	p = 0.366	p = 0.938	p = 0.295
Baby’s weight	r = 0.139	r = 0.033	r = 0.071	r = 0.018
	p = 0.232	p = 0.775	p = 0.549	p = 0.878
Gestational age	rs = 0.057	rs = 0.006	rs = -0.656	rs = -0.519
	p = 0.625	p = 0.960	p ≤ 0.01**	p ≤ 0.01**
Gestational age at delivery	rs = 0.237	rs = 0.395	rs = 0.257	rs = 0.226
	p = 0.039*	p ≤ 0.01**	p = 0.027*	p = 0.053
Interval	rs = 0.045	rs = 0.190	rs = 0.710	rs = 0.652
	p = 0.700	p = 0.103	p ≤ 0.01**	p ≤ 0.01**

The transfer ratio was divided into the following two categories: < 1 and ≥ 1. In the vaccinated group, 64.5% had anti-nucleocapsid (anti-N) transfer ratios ≥ 1, and 60.5% had anti-spike (anti-S) transfer ratios ≥ 1. In the COVID-19-infected group, 50% had an anti-N transfer ratio < 1, and 60.8 % had an anti-S transfer ratio < 1. Frequencies of transfer ratios < 1 and ≥ 1, stratified by maternal factors are shown in Table 4 for the vaccinated group and COVID-19-infected group.

**Table 4:** Frequencies and association of maternal factors with antibody transfer ratios of vaccinated and COVID-19-infected patients are presented as N (%). *Significant p-value < 0.05. P-value was calculated using chi-square test or Fisher’s exact test as appropriate. COVID-19: coronavirus disease 2019; anti-N: anti-nucleocapsid; anti-S: anti-spike

Variables			Anti-N transfer ratio		p-Value	Anti-S transfer ratio		p-Value
			< 1	≥ 1		< 1	≥ 1	
Vaccinated mothers and cord blood	Mother’s age	18-25	7 (30.4)	16 (69.6)	0.044*	8 (34.8)	15 (65.2)	0.645
		26-35	11 (28.2)	28 (71.8)		15 (38.5)	24 (61.5)	
		> 35	9 (64.3)	5 (35.7)		7 (50)	7 (50)	
	BMI	< 18.5 (underweight)	0	1 (100)	0.983	0	1 (100)	0.509
		18.5-25 (normal)	2 (33.3)	4 (66.7)		2 (33.3)	4 (66.7)	
		26-30 (overweight)	10 (35.7)	18 (64.3)		9 (32.1)	19 (67.9)	
		31-35 (obese grade 1)	6 (33.3)	12 (66.7)		8 (44.4)	10 (55.6)	
		36-40 (obese grade 2)	6 (40)	9 (60)		9 (60)	6 (40)	
		> 40 (obese grade 3)	2 (50)	2 (50)		1 (25)	3 (75)	
	Gender	Female	16 (40)	24 (60)	0.390	14 (35)	26 (65)	0.400
		Male	11 (30.6)	25 (69.4)		16 (44.4)	20 (55.6)	
	Baby’s weight (g)	< 2500	6 (75)	2 (25)	0.061	6 (75)	2 (25)	0.05*
		2500-3500	13 (29.5)	31 (70.5)		18 (40.9)	26 (59.1)	
		> 3500	8 (33.3)	16 (66.7)		6 (25)	18 (75)	
	Delivery gestational age (weeks)	≤ 37 weeks	12 (57.1)	9 (42.9)	0.015*	14 (66.7)	7 (33.3)	0.003*
		> 37 weeks	15 (27.3)	40 (72.7)		16 (29.1)	39 (70.9)	
	Interval	0-50	8 (38.1)	13 (61.9)	0.670	10 (47.6)	11 (52.4)	0.547
		51-100	12 (41.4)	17 (58.6)		12 (41.4)	17 (58.6)	
		101-150	6 (26.1)	17 (73.9)		8 (34.8)	15 (65.2)	
		> 150	1 (50)	1 (50)		0	2 (100)	
Covid-19-infected mothers and cord blood	Mother’s age	18-25	13 (46.4)	15 (53.6)	0.788	16 (57.1)	12 (42.9)	0.817
		26-35	17 (50)	17 (50)		22 (64.7)	12 (35.3)	
		> 35	7 (58.3)	5 (41.7)		7 (58.3)	5 (41.7)	
	BMI	18.5-25 (normal)	5 (45.5)	6 (54.5)	0.445	8 (72.7)	3 (27.3)	0.408
		26-30 (overweight)	15 (48.4)	16 (51.6)		21 (67.7)	10 (32.3)	
		31-35 (obese grade 1)	13 (56.5)	10 (43.5)		11 (47.8)	12 (52.2)	
		36-40 (obese grade 2)	4 (66.7)	2 (33.3)		4 (66.7)	2 (33.3)	
		> 40 (obese grade 3)	0	3 (100)		1 (33.3)	2 (66.7)	
	Gender	Female	14 (45.2)	17 (54.8)	0.480	17 (54.8)	14 (45.2)	0.372
		Male	23 (53.5)	20 (46.5)		28 (65.1)	15 (34.9)	
	Baby’s weight (g)	< 2500	5 (100)	0	0.057	5 (100)	0	0.216
		2500-3500	23 (48.9)	24 (51.1)		27 (57.4)	20 (42.6)	
		> 3500	9 (40.9)	13 (59.1)		13 (59.1)	9 (40.9)	
	Delivery gestational age (weeks)	≤ 37 weeks	13 (65)	7 (35)	0.116	18 (90)	2 (10)	0.002*
		> 37 weeks	24 (44.4)	30 (55.6)		27 (50)	27 (50)	
	Interval	0-50	16 (94.1)	1 (5.9)	< 0.01*	16 (94.1)	1 (5.9)	< 0.01*
		51-100	17 (58.6)	12 (41.4)		19 (65.5)	10 (34.5)	
		101-150	3 (17.6)	14 (82.4)		8 (47.1)	9 (52.9)	
		> 150	1 (9.1)	10 (90.9)		2 (18.2)	9 (81.8)	

From vaccinated patients, gestational age at delivery had a statistically significant association with anti-N and anti-S transfer ratios with preterm delivery having a high prevalence with anti-transfer ratios of < 1 and delivery at > 37 weeks having a high prevalence with anti-transfer ratios of ≥ 1 (Table 4). Gestational age at delivery from COVID-19-infected patients had a statistically significant association with anti-S transfer ratio with 90% of the preterm delivery having an anti-S transfer ratio < 1. Interval at COVID-19 infection had a statistically significant association with anti-N and anti-S transfer ratios as interval of < 100 days had a high prevalence in anti-transfer ratios of < 1 and interval of > 100 had a high prevalence in anti-transfer ratios of ≥ 1 (Table 4).

Difference in the sequelae of both vaccinated and COVID-19-infected preterm, still-born, and preeclampsia babies are represented in Table 5. There were no statistically significant differences between the two groups' sequelae.

**Table 5:** Difference of sequelae of both vaccinated and COVID-19-infected patients. P-value was calculated by two sample proportion (Fisher’s exact test). COVID-19: coronavirus disease 2019

Variables	Vaccinated (n=80)	COVID-19 infected (n=74)	p-Value
Preterm	8	15	0.112
Still-born	0	1	0.481
Preeclampsia	2	4	0.428

**Figure 1:**
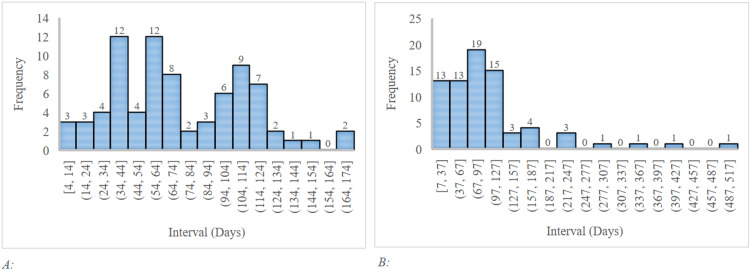
(A) Interval frequencies of antibodies from second dose of vaccination. (B) Interval frequencies of antibodies from COVID-19 infection. COVID-19: coronavirus disease 2019

Antibody intervals frequency of the vaccinated group from the second dose was highest within 34-44 days and 54-64 days, whereas intervals from COVID-19 infection were highest within 67-97 days (Figures 1, panels A and B).

**Figure 2:**
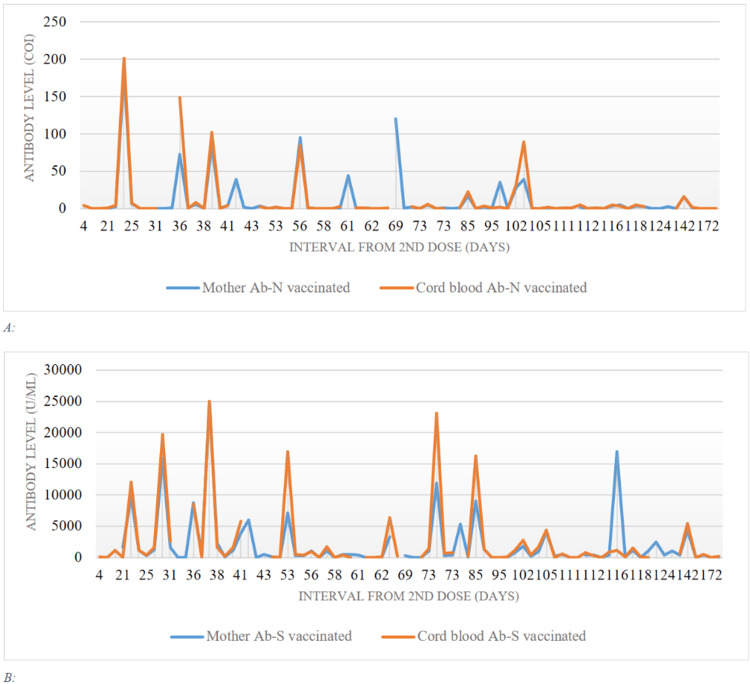
(A) Comparison between antibody N of vaccinated mothers and cord blood. (B) Comparison between antibody S of vaccinated mothers and cord blood.

Mother's and cord blood antibodies N levels peaked 21 days after the second dose and then gradually decreases over time. Both mother's and cord blood antibodies N were at their lowest level starting from 105 days after the second dose (Figure 2, panel A). After 37 days from the second dose, the mother's and cord blood antibodies-S levels were both at their highest level (25000 U/mL). Antibodies S levels were < 1000 U/mL at 56-73 days following the second dose. Except for one vaccinated mother who had an antibody S of (16907 U/mL) after 95 days, both mothers and cord blood antibodies S were at their lowest levels (Figure 2, panel B). 

**Figure 3:**
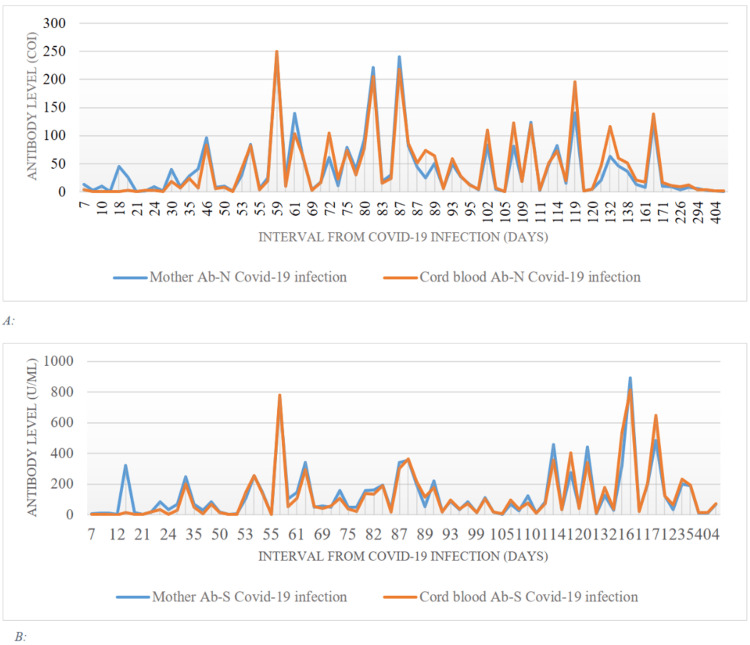
(A) Comparison between antibody N of COVID-19-infected mothers and cord blood. (B) Comparison between antibody S of COVID-19-infected mothers and cord blood. COVID-19: coronavirus disease 2019

From 46 to 171 days following COVID-19 infection, mothers' and cord blood antibodies N levels varied between 100 and 250 COI levels (Figure 3, panel A). Antibodies S levels in mothers and cord blood rise to levels above 400 U/mL, starting from 101 days after COVID-19 infection. One mother and cord blood antibodies S were above 700 U/mL, 59 days after the infection (Figure 3, panel B).

**Figure 4:**
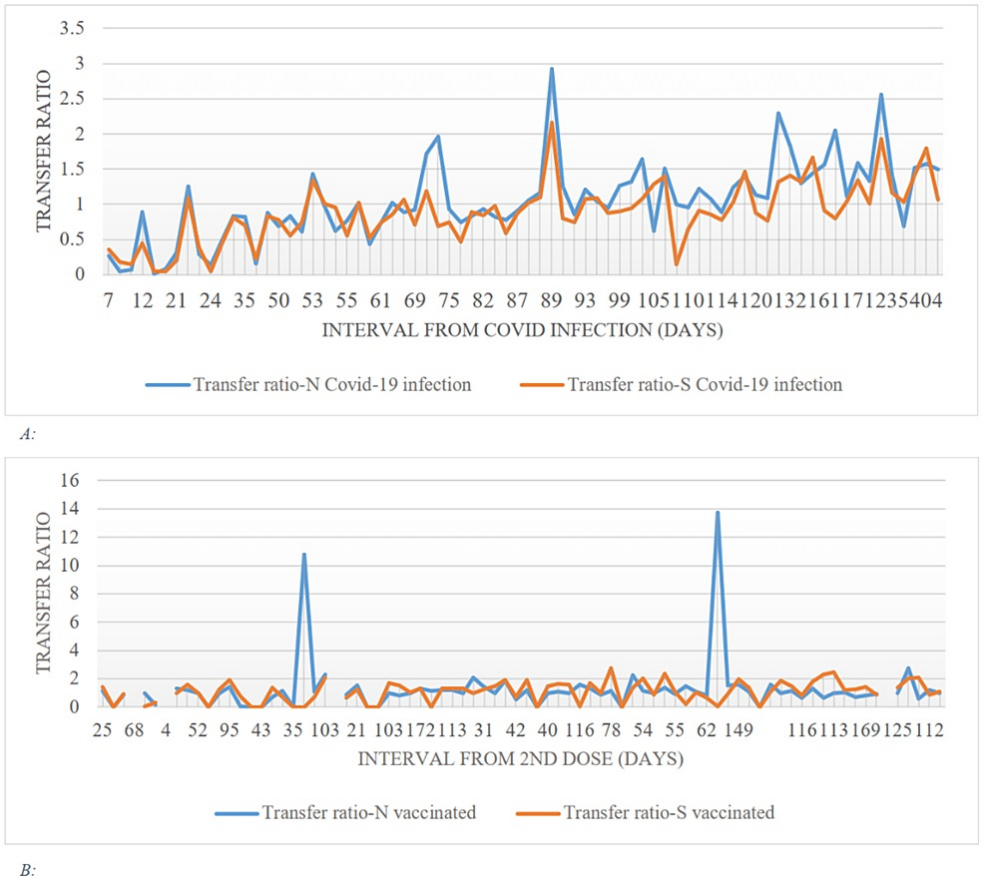
(A) Comparison between antibody's transfer ratios of COVID-19-infected mothers and cord blood. (B) Comparison between antibody's transfer ratios of vaccinated mothers and cord blood. COVID-19: coronavirus disease 2019

Figure 4, panel A, illustrates how the transfer ratios for both mothers and cord blood rise as the number of days since COVID-19 infection increases. The transfer ratios of mothers and cord blood rise as the number of days following the second dose increases, with two patients having high transfer ratios of 10.75 after 21 days and 13.76 after 60 days (Figure 4, panel B).

## Discussion

The COVID-19 pandemic raised several questions about pregnant women, including the impact of infection during pregnancy and transmission of the disease to the newborn. Protection of pregnant women and fetuses against the worst of the disease remains an enormous concern for obstetricians. Immune responses to the infection and vertical transmission of antibodies are accounted for when giving the vaccination during pregnancy. Immunization is optimized for maternal and infant protection. Elements that affect vaccine acceptance are public awareness of infection risk, vaccine safety, and the method of public communication regarding the vaccine and its safety [6]. Our populations were encouraged to get Sinophram and Pfizer.

Group 1 patients' age range was 19-42 years, and group 2 patients' age range was 18-42 years. The populations were of reproductive age, and titers varied along with maternal age. Immunity decreased as age increased [7]. Antibody S had a weak negative correlation with maternal age while, surprisingly, antibody N had a positive correlation with age. Individual variations in response to immunization and immunological reactions to COVID-19 infection occurred at the reproductive ages. A similar study by Yang et al. (2021) showed differences in the immune response in different age categories of the general population [8]. Collier et al. found a better response in vaccinated younger people than in older people of the general population [9]. Obesity is a risk factor for COVID-19 and could cause a stronger immune response. Our study grouped titers of antibodies and the transfer ratio and did not find any association with BMI. By contrast, Soffer et al. (2021) identified a relationship between COVID-19 antibodies and obesity in the general population [10]. In our study, four vaccinated mothers and 21 COVID-19 patients had comorbidities such as hypothyroidism, gestational diabetes, and bronchial asthma.

A study by Prabhu et al. (2021) found that pregnant women who received mRNA vaccines produced maternal antibodies as early as five days after their first vaccination dose. Notably, passive immunity via the placenta to the neonate occurred as early as 16 days after the first vaccination dose [11]. In our study, the first dose received was at the gestational age of 26 weeks, and the second dose was at 29 weeks. The reported average gestational age at the onset of COVID-19 infection was 26 weeks. An analysis of antibodies was conducted at the time of delivery. The frequency of antibody intervals in the vaccinated group from the second dose was highest from 34 to 44 days and 54 to 64 days whereas intervals from COVID-19 infection were highest between 67 and 97 days. Comparison of antibody titers between the groups showed statistically significant, higher levels of anti-S and vertical transmission in the vaccinated population and anti-N in the infected population. This denotes the higher immunogenic response to the spike protein that occurred in the vaccinated group and the higher immunogenic response to the nucleocapsid protein that occurred in the COVID-19-infected group. Supporting our study, immune responses in the mother and transmission to the fetus of the mRNA vaccines were more elevated in the vaccinated mothers than in the COVID-19-recovered mothers [12]. A study by Polack et al. (2020) showed that two doses of the BioNTech/Pfizer BNT162b2 mRNA vaccine gave 95% protection against COVID-19 in the non-pregnant age group of 16 years or more [13]. In our study, the mean gestational age at vaccination was 29 weeks, and all of the vaccinated population included in our study received two doses of the vaccine. They had protection until the early postpartum period, which supports the use of the vaccine to protect pregnant women from the virus during the pregnancy, which is a more critical period. However, the vaccine’s long-term protection requires further study. The levels of protection of the COVID-19 group and the vaccinated group remain inconclusive. Nevertheless, vaccinating the pregnant population is important because it reduces the severity and transmission of the disease [14,15]. Vitiello (2021) reported that there was decreased transmission of the virus among the vaccinated population. The level of protection varies after each dose of the vaccine and according to the variant [16]. Supporting this view, our study showed statistically significant levels of anti-S and a higher transfer ratio in the vaccinated population compared with the COVID-19-infected, non-vaccinated population. Vaccination could reduce the rate of COVID-19 infection in pregnant women as well as in neonates.

In our study, the latency period was calculated either from the second dose of the vaccine or from the diagnosis of COVID-19 infection to delivery. The latency period showed statistical significance for both anti-N and anti-S titers and transfer ratios for the COVID-19-infected population. Higher transfer ratios occurred when the interval was greater than 100 days. A study by Mithal et al. demonstrated that vaccinated women had higher transfer ratios of antibodies according to their latency period [17]. Yang et al. showed that the level of vertical transmission of antibodies was elevated at the time of delivery in vaccinated women, regardless of the timing of vaccination [18]. Edlow et al.’s study compared the vertical transmission of different types of viral antibodies and reported decreased transfer of SARS-CoV-2 antibodies; thus, COVID-19 infection in neonates and infants from infected mothers was predicted [19]. A study by Flannery et al. showed that an immunological response and vertical transfer of antibodies predicted potential protection against infection in pregnant women and neonates [20]. However, higher maternal levels of anti-S and transfers through placentae following vaccination could provide more protection against the virus. In our study, the transfer ratio of anti-S was calculated at 1.23 (0.68, 1.6) in the vaccinated group and 0.88 (0.68,1.08) in the COVID-19-recovered group. In an exploratory, descriptive, and prospective cohort study, Collier et al. concluded that the COVID-19 mRNA vaccine was immunogenic and evoked antibodies that were transferred to cord blood and breast milk [21]. This is consistent with our study with regard to cord blood transfer. Vaccination (mRNA) during the antenatal period induces a humoral response in the mother, which is transferred to the fetus. This reinforces the advantage of vaccination during pregnancy, a conclusion reached by Beharier et al. [22].

A prospective cohort study by Nir et al. involving 64 parturient, vaccinated women and 11 parturient women who contracted COVID-19 during pregnancy was conducted to identify the transfer ratio of SARS-CoV-2 antibodies from mother to neonate. They found that transfer of SARS-CoV-2 antibodies across the placenta occurred more frequently in pregnant women vaccinated with the BNT162b2 mRNA vaccine. This was confirmed by the positive level of antibodies in both maternal serum and cord blood. The vaccine proved to have a double advantage - maternal protection and neonatal humoral immunity [23].

Our study showed potent immune responses and antibody transfer to the fetus in both group 1 and group 2. The antibody transfer ratio showed significant correlation between gestational age and delivery in group 1 whereas group 2 showed a correlation between gestational age and the latency period. The transfer ratio was divided into subcategories according to the levels, either more than one or less than one. Both group 1 and group 2 showed that the level of transfer ratios was higher at a gestational age of more than 37 weeks. Comparable antibody transfer ratios in both groups increased according to the maturity of the fetus. Mithal et al. showed that the COVID-19 mRNA vaccine given during the third trimester generated similar antibody levels between mother and fetus, which were greater in the case of COVID-19 infection [17]. Beharier et al. reported a similar immune response to vaccination and COVID-19 disease and showed that the IgG transfer ratio was higher during the second trimester than the third [22]. The level of transfer ratios was influenced by the birth weight of the infant. In group 1, as the baby's weight decreased, the anti-S transfer ratio decreased as well (a weight of < 2500 g had 75% prevalence of an anti-S transfer ratio < 1). As the baby's weight increased, the anti-S transfer ratio also increased (a weight of > 3500 g had 75% prevalence of an anti-S transfer ratio ≥ 1). There was no significant correlation between transfer ratio and the gender of the infant.

Additionally, we compared the incidence of obstetric complications such as fetal growth restriction, preeclampsia, stillbirth, and preterm delivery in the vaccinated group to that of the COVID-19-infected group. Although the frequency of complications was higher in the COVID-19-infected group than in the vaccinated group, no statistical significance was found. The direct relationship between the complications and disease could not be confirmed. Clinical trials have proven the safety of the vaccine in general; however, trials and data regarding pregnant women are limited [4,24]. Principi et al. reported the safety outline of the vaccine [15]. Shimabukuro et al. revealed that the vaccine’s side effects are mainly fever, headache, body pain, and chills and recommended more studies focused on perinatal outcomes [25]. Beharier et al. reported the rate of preterm delivery, stillbirth, and preeclampsia in infected pregnant women [12]. No concerning issues were observed by Trostle et al. in pregnant women who received the mRNA COVID-19 vaccine [26]. Peretz et al. deduced that no preterm deliveries occurred among the 57 vaccinated women who delivered [27]. In our study, compared with the vaccinated group, the COVID-19-infected group had a higher rate of preterm delivery (eight vs. 15), stillbirth (zero vs. one), and preeclampsia (two vs. four). Our study illustrated immune responses to vaccination and COVID-19 infection as well as placental transfer of the antibodies. We also included the sequelae of group 1 and group 2 in our study to reassure our pregnant population.

Why was this study conducted?

The study of immune responses, vertical transmission, and effects of the COVID-19 infection and vaccination is needed to reduce the impact of COVID-19-related morbidities and mortalities in pregnant women.

Key findings

During pregnancy, although vaccination and COVID-19 disease produced the immune responses along with the vertical transmission, there was a significant antibody transfer to neonates. A higher response of anti-S and transfer ratio of anti-S occurred in the vaccinated group, which is likely protective for the mother and newborn. Antibody titers varied according to maternal and gestational age. Latency period was significant in the COVID-19-infected population whereas birth weight of the infant was significant in the vaccinated population.

What does this add to science?

All of our study population developed a reasonable immune response and had vertical transmission. The safety of the vaccine is reassuring and promising for pregnant women. Additionally, vaccinated pregnant women were effectively protected from COVID-19 infection during pregnancy and transferred their antibodies to neonates. Antibody titers varied in different age groups. The transfer ratio was higher in term fetuses. The maternal BMI has no correlation with antibody titers or transfer ratios. The infants’ gender had no association with transfer ratios.

Clinical and research implications

The reassurance of vaccination without major side effects during pregnancy and protection from COVID-19 helped our pregnant women cope with current modifications in their lifestyles resulting from the COVID-19 pandemic. Even though immunity is helpful in overcoming the current pandemic, further studies of waning antibody levels, reoccurrence of the disease, and duration of protection will support recommendation or refutation of booster vaccine doses.

Strength of the study

In our study, vaccinated participants were not infected with COVID-19, and the virus was excluded using nasopharyngeal samples using the polymerase chain reaction (PCR) or SARS-CoV-2 GeneXpert before checking antibody levels. The infected population was not vaccinated either before or during pregnancy. These criteria added clarity to the immune response of the groups. The correlation of antibodies to transfer ratios was calculated in the infected and vaccinated groups in relation to maternal demographic variables such as maternal age, BMI and gestational age, gestational age at delivery, gender of the infant, and birth weight of the infant. Transfer ratios were divided into different ranges, and correlation was calculated considering maternal and clinical factors. Latency period was divided into different ranges, and correlation was calculated considering transfer ratios.

Limitations

The sample size is small in both the COVID-19-infected and vaccinated groups. Our study did not analyze the post-delivery sequences for the mother and newborn. The waning of antibody levels was not analyzed and requires further study. Categorization of IgG and IgM was not completed in our analysis.

## Conclusions

The pregnant women generated anti-N and S in response to COVID-19 infection and vaccination and efficiently transferred these to neonates. The immune response to COVID-19 infection and vaccination was individualized, and titers of anti-N were higher in COVID-19-recovered women. Levels of anti-S and transfer ratios of anti-S were higher in the vaccinated pregnant women. Antibody variations occurred based on maternal age in both groups. BMI showed no association with antibody titers or transfer ratios. Transfer ratios were higher in term babies in both groups and showed no significant relation to the baby’s gender. A prolonged latency period had a higher transfer ratio in the COVID-19-infected group whereas the birth weight of the infants was significant in the vaccinated pregnant population. However, the level of immunity and duration of protection remain inconclusive. Both COVID-19-recovered and vaccinated women showed an immune response as well as a transfer of antibodies at different rates to neonates. Further studies could include the risk of COVID-19 severity during pregnancy vs. the benefit of vaccination and which group gives more protection to neonates against COVID-19 and future possible variants.

## References

[1] DeBolt CA, Bianco A, Limaye MA (2021). Pregnant women with severe or critical coronavirus disease 2019 have increased composite morbidity compared with nonpregnant matched controls. Am J Obstet Gynecol.

[2] Jering KS, Claggett BL, Cunningham JW, Rosenthal N, Vardeny O, Greene MF, Solomon SD (2021). Clinical characteristics and outcomes of hospitalized women giving birth with and without COVID-19. JAMA Intern Med.

[3] Knight M, Bunch K, Vousden N (2020). Characteristics and outcomes of pregnant women admitted to hospital with confirmed SARS-CoV-2 infection in UK: national population based cohort study. BMJ.

[4] Royal College of Obstetricians and Gynecologists. (2022). Coronavirus (COVID-19), infection in pregnancy. https://www.rcog.org.uk/media/xsubnsma/2022-03-07-coronavirus-covid-19-infection-in-pregnancy-v15.pdf..

[5] (2022). Use of COVID-19 vaccines in the United States. https://www.cdc.gov/vaccines/covid-19/clinical-considerations/covid-19-vaccines-us.html.

[6] Januszek SM, Faryniak-Zuzak A, Barnaś E (2021). The approach of pregnant women to vaccination based on a COVID-19 systematic review. Medicina (Kaunas).

[7] Bartleson JM, Radenkovic D, Covarrubias AJ, Furman D, Winer DA, Verdin E (2021). SARS-CoV-2, COVID-19 and the ageing immune system. Nat Aging.

[8] Yang HS, Costa V, Racine-Brzostek SE (2021). Association of age with SARS-CoV-2 antibody response. JAMA Netw Open.

[9] Collier DA, Ferreira IA, Kotagiri P (2021). Age-related immune response heterogeneity to SARS-CoV-2 vaccine BNT162b2. Nature.

[10] Soffer S, Glicksberg BS, Zimlichman E (2021). The association between obesity and peak antibody titer response in COVID-19 infection. Obesity (Silver Spring).

[11] Prabhu M, Murphy EA, Sukhu AC (2021). Antibody response to coronavirus disease 2019 (COVID-19) messenger RNA vaccination in pregnant women and transplacental passage into cord blood. Obstet Gynecol.

[12] Beharier O, Plitman Mayo R, Raz T (2021). Efficient maternal to neonatal transfer of antibodies against SARS-CoV-2 and BNT162b2 mRNA COVID-19 vaccine. J Clin Invest.

[13] Polack FP, Thomas SJ, Kitchin N (2020). Safety and efficacy of the BNT162b2 mRNA COVID-19 vaccine. N Engl J Med.

[14] Huang YZ, Kuan CC (2022). Vaccination to reduce severe COVID-19 and mortality in COVID-19 patients: a systematic review and meta-analysis. Eur Rev Med Pharmacol Sci.

[15] Principi N, Esposito S (2021). Is the immunization of pregnant women against COVID-19 justified?. Vaccines (Basel).

[16] Vitiello A, Ferrara F, Troiano V, La Porta R (2021). COVID-19 vaccines and decreased transmission of SARS-CoV-2. Inflammopharmacology.

[17] Mithal LB, Otero S, Shanes ED, Goldstein JA, Miller ES (2021). Cord blood antibodies following maternal coronavirus disease 2019 vaccination during pregnancy. Am J Obstet Gynecol.

[18] Yang YJ, Murphy EA, Singh S (2022). Association of gestational age at coronavirus disease 2019 (COVID-19) vaccination, history of severe acute respiratory syndrome coronavirus 2 (SARS-CoV-2) infection, and a vaccine booster dose with maternal and umbilical cord antibody levels at delivery. Obstet Gynecol.

[19] Edlow AG, Li JZ, Collier AY (2020). Assessment of maternal and neonatal SARS-CoV-2 viral load, transplacental antibody transfer, and placental pathology in pregnancies during the COVID-19 pandemic. JAMA Netw Open.

[20] Flannery DD, Gouma S, Dhudasia MB (2021). Assessment of maternal and neonatal cord blood SARS-CoV-2 antibodies and placental transfer ratios. JAMA Pediatr.

[21] Collier AY, McMahan K, Yu J (2021). Immunogenicity of COVID-19 mRNA vaccines in pregnant and lactating women. JAMA.

[22] Beharier O, Mayo RP, Raz T (2021). Efficient maternal to neonatal transfer of antibodies against SARS-CoV-2 and BNT162b2 mRNA COVID-19 vaccine. J Clin Invest.

[23] Nir O, Schwartz A, Toussia-Cohen S (2022). Maternal-neonatal transfer of SARS-CoV-2 immunoglobulin G antibodies among parturient women treated with BNT162b2 messenger RNA vaccine during pregnancy. Am J Obstet Gynecol MFM.

[24] Gill L, Jones CW (2021). Severe acute respiratory syndrome coronavirus 2 (SARS-CoV-2) antibodies in neonatal cord blood after vaccination in pregnancy. Obstet Gynecol.

[25] Shimabukuro TT, Kim SY, Myers TR (2021). Preliminary findings of mRNA COVID-19 vaccine safety in pregnant persons. N Engl J Med.

[26] Trostle ME, Limaye MA, Avtushka V, Lighter JL, Penfield CA, Roman AS (2021). COVID-19 vaccination in pregnancy: early experience from a single institution. Am J Obstet Gynecol MFM.

[27] Peretz SB, Regev N, Novick L (2021). Short-term outcome of pregnant women vaccinated with BNT162b2 mRNA COVID-19 vaccine. Ultrasound Obstet Gynecol.

